# VISTO: An open-source device to measure exposure time in psychological experiments

**DOI:** 10.1016/j.mex.2021.101427

**Published:** 2021-06-22

**Authors:** Andrea De Cesarei, Michele Marzocchi, Geoffrey R. Loftus

**Affiliations:** a Department of Psychology, University of Bologna, Italy; b Department of Psychology, University of Washington, United States

**Keywords:** Visual processing, Exposure time, Masking, Device synchronization

## Abstract

The study of higher cognitive processes often relies on the manipulation of bottom-up stimulus characteristics such as exposure time. While several software exist that can schedule the onset and offset time of a visual stimulus, the actual exposure time depends on several factors that are not easy to control, resulting in undesired variability within and across studies. Here we present VISTO, a simple device built on the Arduino platform that allows one to measure the exact onset and offset of a visual stimulus, and to test its synchronization with a trigger signal. The device is used to measure the profile of luminance waveforms in arbitrary analog/digital (AD) units, and the implications of these luminance profiles are discussed based on a model of information accumulation from visual exposure. Moreover, VISTO can be calibrated to match the brightness of each experimental monitor. VISTO allows for control of stimulus timing presentation, both in classical laboratory settings and in more complex settings as technology allows to use new display devices or acquisition equipment. In sum, VISTO allows one to:

• measure the profile of luminance curves.
• determine the exposure time of a visual stimulus.
• measure the synchronization between a trigger signal and a visual stimulus.

## Graphical abstract

**Figure:**
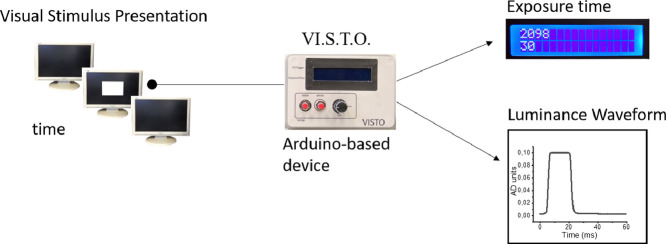

Specifications table

**Table:** 

Subject Area:	Psychology
More specific subject area:	*Visual Cognition*
Method name:	*Arduino Platform, electronic parts*
Name and reference of original method:	N.A
Resource availability:	*Arduino Board, electronic parts, free code given in the Supplementary material*

## Method details

Central to most accounts of vision is the idea that visual perception depends on the acquisition of information from the environment, and that acquired information is processed in a sequence of increasingly complex stages. Information acquisition is central to theories of vision in computational neuroscience (e.g., [1]), and cognitive science (e.g., [14]). In experimental settings, the acquisition of information is manipulated by controlling the type (e.g., global or local; [8]), the strength (e.g., contrast) and the time (exposure time, masking) with which a stimulus is available for processing.

Manipulation of exposure time as a means of modulating information acquisition has been used in several experimental paradigms that investigated picture memory [20], face and scene recognition [22], iconic memory [28], attention [21], and emotional response [5]. In all these cases, it was presumed that longer exposure times would result in more acquired information and thereby more accurate responses.

While the manipulation of exposure time is highly informative concerning several aspects of cognition, achieving precise presentation timing in the laboratory is not straightforward for several reasons. First, most operating systems are not real-time systems, meaning that several programs are run at once with different priority. This means that the timing of a specific program (e.g., stimulus display) may easily be influenced by a simultaneously running system program — for instance, system software like cloud syncing, antivirus or system checks are running concurrently with the experiment — and thus may unpredictably alter the timing of the experiment. Second, and most relevant to the present paper, the devices used to present visual stimulation (e.g., video cards, monitors and projectors) differ in terms of technology and performance. Popular display devices include slide projectors, LCD projectors, LCD monitors, CRT monitors, TFT monitors, each of which entails different benefits and caveats. For instance, slide projectors and CRT displays were shown to have an excellent accuracy in the timing of stimulus onset and offset [12,^31^]; however the acoustic noise of mechanical shutters and the magnetic interference of CRT monitors may interfere with specific aspects of the experimental presentation (e.g., by causing an orienting to the acoustic shutter noise or by interfering with fMRI recording). On the other hand, recent LCD displays have been shown to have good properties in terms of stimulus visualization, persistence, and timing [11,13]. To further complicate matters, some experiments require the synchronization of two (or more) devices. Here, we describe a simple device that can be used in any laboratory to estimate the timing properties (onset, offset, duration) of stimulus display. We dubbed this device “VI.S.T.O.” (VIsual Stimulus Temporal Onset, “seen” in Italian).

LCD monitors display images beginning from one edge of the screen and progressively moving through the remaining portion of the screen. After the entire display has been plotted, drawing of the next display begins. The speed of this process defines the *refresh rate*, which may vary from slower (e.g., 60 frames per second) to faster (up to 200 frames per second). The minimum achievable exposure time depends on the refresh rate of the apparatus. For instance, in the case of a 60 Hz monitor, each frame lasts 1,000/60 = 16.67 ms; thus, the minimal presentation time that will be achieved with such a monitor is 16.67 ms, and it can be increased only in increments of 16.67 ms. By contrast, with a 200 Hz monitor, each frame lasts 1,000/200 = 5 ms; thus, the minimal presentation time is 5 ms, and can be increased in increments of 5 ms.

In addition to refresh rate, display devices differ in the capability to switch from one image luminance (e.g., a black screen) to a different image luminance (e.g., a white patch). The manner in which the display luminance rises (in the case of a change from dark to light) or falls (in the opposite case) may differ substantially, depending on the display technology. As a result, actual luminance profiles can be characterized by slopes which vary in steepness both as they rise (black to white) and/or as they fall (white to black) and may substantially depart from an ideal rectangular waveform, which would be defined by 0 ms rise and fall times.

Finally, experimenters are often interested in acquiring data from one acquisition system (e.g., remote or wearable eye tracker, wearable devices, EEG systems), while using a different device for stimulus presentation (e.g., virtual reality, laboratory monitor, or similar). In all these cases, it is critical that acquisition and presentation devices are well synchronized with each other, in order to observe phenomena that are time-locked to an external event such as Event-Related Potentials (ERPs), saccade onsets, and so on.

### Motivation for the present Method

Achieving precise timing is critical in studies investigating information acquisition from briefly presented stimuli. However, several difficulties, including the ones described above, can affect experimental timing. Accordingly, both commercial products and open source devices have been developed to serve specific needs; these include the BlackBox Toolkit [17,18], and the Schultz Cigarette Burn Toolbox (SCiBuT; [27]). Moreover, other devices have been devoted to collect both triggers and responses from response pads and other devices, while keeping each in sync with the others ([7], [25],^26^,32]; see also Trigger Station by BrainTrends Ltd.). To check the timing of visual stimulus presentation, several laboratories have developed in-house routines to monitor accurate timing in exposure time; these rely on instruments (e.g., oscilloscopes, digital amplifiers; e.g., [19]) that are specific to each lab. Here instead, we present VISTO, a simple but general tool that can be built based on the open source, low cost, and widely available Arduino programming board [6], and few electronic parts. The primary application of VISTO is prior to running an experiment, when the software and hardware have to be set up to obtain the desired exposure times. However, VISTO can also be employed during an experiment to detect and log the onset of visual markers which can then be used during data analysis. We designed VISTO such that it could be able to 1) measure the profile of the luminance waveform following stimulus presentation, and export these data for later analysis, and 2) report on-line the exposure time of a stimulus.

The remainder of this article comprises two main sections. In the first, we use VISTO to collect luminance profiles, in arbitrary AD units, for stimuli of varying exposure times. These profiles are then compared with an ideal rectangular luminance profile, based on quantitative theories of visual perception that allow quantitative predictions regarding the acquisition of information from a visual stimulus. In the second section, we describe how to measure exposure time and display it in real time. In each section, we provide specific instructions for how to use VISTO to perform each of these analyses.

## Methods

### Material

Altogether, the price of all materials at the time of building the prototype was about 60 €. The following materials were used:

• Arduino board mod. Arduino Mega 2560.
• Photodiode mod. BPW42.
• Potentiometer 500 Kohm.
• 25 pin (LPT-type) female plug.
• LCD Panel 16 × 2 compatible Hitachi HD44780.
• cables, tin, and soldering iron.

**Fig. 1:**
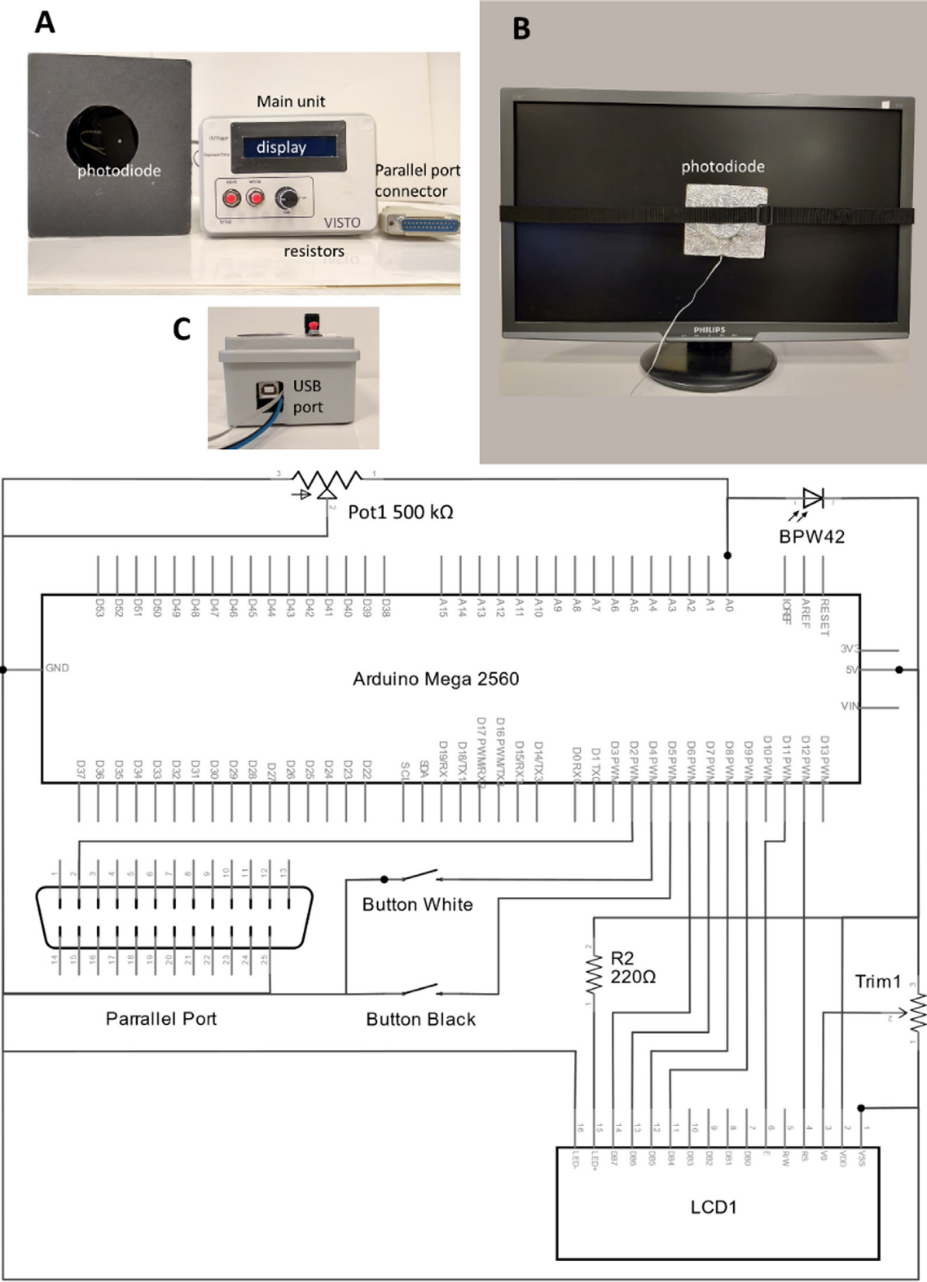
A–C: VISTO main components. D: diagram of the electronic circuit.

The circuit diagram is displayed in Fig. 1. The photodiode is placed inside a container that can be then attached to the monitor. The container is shielded from light on all sides, so that only light coming from the display device can activate the photodiode, while all other light sources are blocked out. The photodiode signal is then conveyed to an adjustable potentiometer (up to 500 KOhm), which allows the experimenter to adjust data acquisition to monitors that differ in luminance range. The Arduino board can digitally acquire data at a high sample rate (here, >=2000 Hz) at 8-bit precision. When a trigger from the parallel port is used to synchronize two devices, activation of pin 2 (binary code 00000010) starts the acquisition of the luminance profile, or the measurement of the trigger-display synchronization.

### Luminance Waveform Acquisition

The first aim of VISTO was to acquire luminance values from visual stimuli, to describe the luminance profiles of each stimulus as displayed on the experimental monitor. Our goal was to determine the profile in terms of duration, rising and falling slope of the luminance profile, but not the exact luminance values, e.g. in lux.

### Data Acquisition

Data were acquired at a fixed sampling rate of 5000 Hz and an 8-bit resolution. These parameters were chosen to maximize the amount of data that could be collected for a single stimulus of up to 400 ms duration. The data collected by Arduino were sent in text mode through the serial USB port to an acquisition PC, where it was saved for later analysis.

### Procedure

*Stimuli*. On an LCD monitor (ASUS VG245HE) with a refresh rate set at 60 Hz, uniform black or white patches were displayed for variable amounts of time. Data acquisition at each trial began with a parallel trigger signal. Then, a uniform white patch was presented for a variable time between 16.67 and 150 ms, in 16.67 ms increments. The onset and offset of the picture were synchronized to the beginning of a vertical cycle on the monitor. The experiment was run using E-Prime 2.0 [24].

### Luminance waveform acquisition procedure

Below are the steps needed to calculate the luminance profiles for a stimulus display and transfer it to a dedicated PC.

1) Install Arduino software from www.arduino.cc and connect VISTO to a USB port. Launch the Arduino Ide app, connect the Arduino device to the USB port, and set the Arduino model (menu Tools/Board) to “Arduino Mega 2560”. When this is done, open the sketch file “Profiles.ino” (Supplementary Code A) and load it into the Arduino device (menu Sketch/Load). If the file is loaded correctly, then the device will display the message “PROFILES Ready!”.
2) On the PC to which VISTO is attached, download and extract CoolTermWin (https://freeware.the-meiers.org/). In the extracted folder, open the file baudrates.ini and add the line 1000000, to allow for baudrate = 1,000,000. Run CoolTerm.exe and set baudrate to 1000000 from the menu Connection/Options.
3) Set the name and path where the data will be saved, from the menu Connection/Capture to Textfile/Start; then start data collection by pressing “Connect”.
4) After this, the presentation program can be run, and data will be saved to the chosen textfile. In the presentation program, each trial must begin with a 2 signal (binary code 00000010) sent to the parallel port. This initiates the data acquisition procedure, and data are acquired at 5000 Hz for 400 ms. After this time, data are sent to the USB port of the connected PC.
5) When the presentation program has finished, close the file from the menu Connection/Capture to Textfile/Stop in CoolTermWin. Collected data can be opened as comma-separated values by any data visualization and plotting software.

### Luminance profiles

**Fig. 2:**
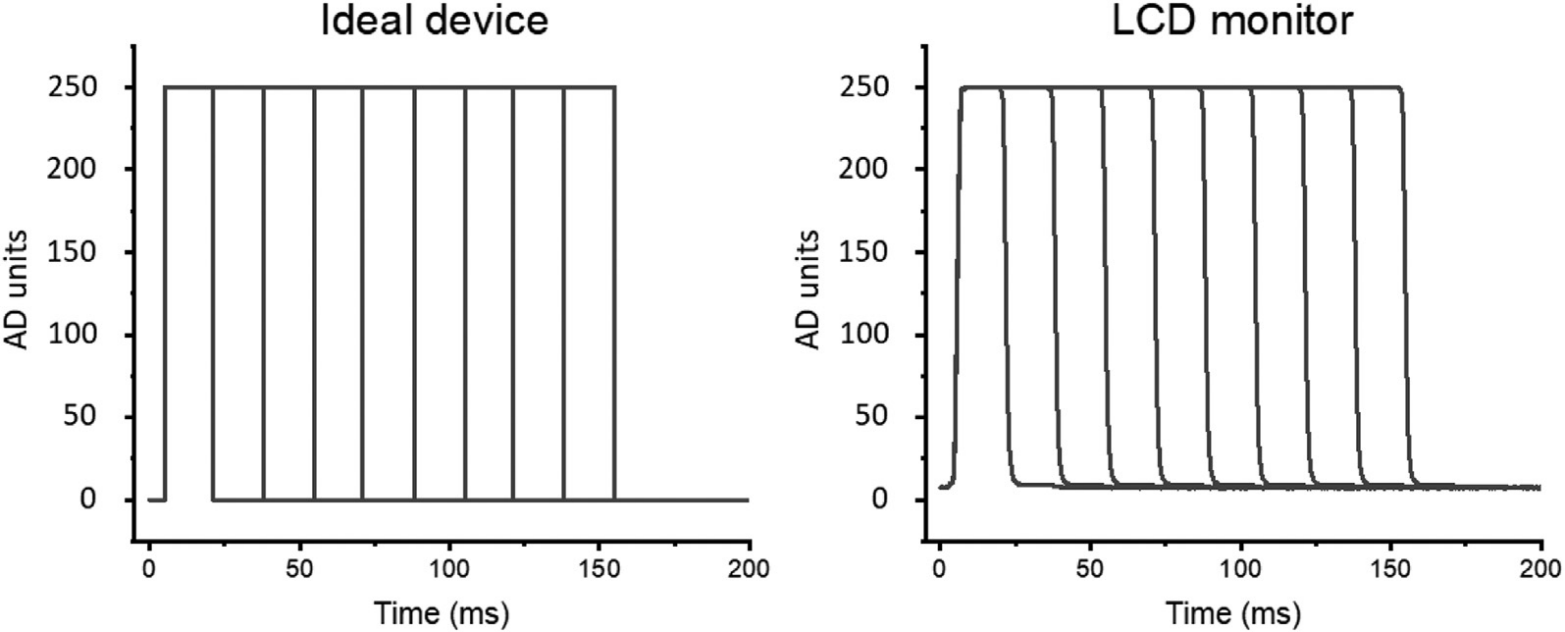
Luminance profiles for an ideal device and an LCD monitor, for stimuli ranging from 16 to 150 ms (each profile includes a 5 ms time before the beginning of the rising portion of the luminance waveform).

Luminance profiles for the ideal rectangular device and the LCD monitor are displayed in Fig. 2.

**Fig. 3:**
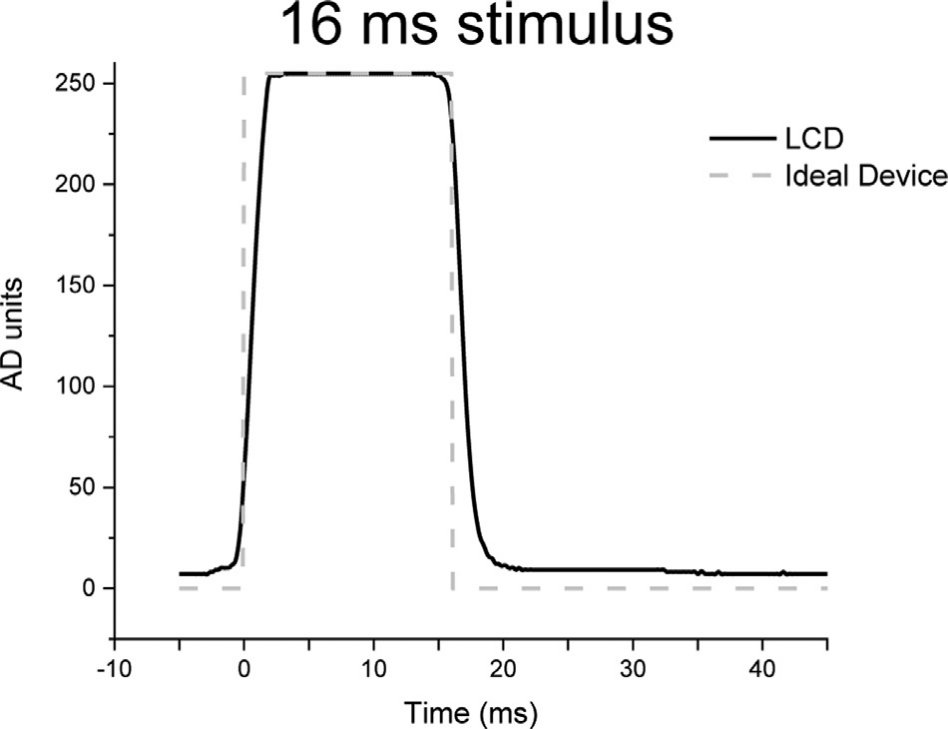
Luminance profiles for an ideal device (rectangular) and an LCD monitor, for a 16 ms stimulus. The LCD waveform has been arbitrarily shifted by 5 ms, to match the onset of the ideal luminance waveform.

While the general pattern of luminance profile did not vary greatly between an ideal and an LCD monitor (Fig. 2), more notable differences appeared with short-duration stimuli such as a 16 ms stimulus (Fig. 3). For a 16 ms stimulus, the real luminance profile had a more sustained luminance and a shallower slope both in its rising and in its falling part compared to the ideal rectangular profile. As a result, an intended 16 ms exposure time resulted in a more extended actual time. While the implications of these differences for actual performance are discussed later, it is important to note that VISTO allows one to precisely measure the luminance profile of each monitor and to adjust experimental settings to achieve the desired timing.

### Implications for theoretical work

When investigating a visual phenomenon, researchers often model the visual input as a constant visual input which is defined by an instantaneous onset and an instantaneous offset. Such a simplifying assumption is usually not met by laboratory devices, which are characterized by small (or large) deviations from a rectangular luminance profile. For instance, it is evident from Fig. 3 that the actual luminance profile is characterized by rising and falling slopes, which are absent in the rectangular waveform and which extend its duration by a few milliseconds.

To what degree should small deviations in luminance profiles from the ideal be construed as worrisome if, in testing some theory, one makes the simplifying assumption that all luminance profiles are rectangular (e.g., [4,^9^,16])? Here we address this issue by contrasting a rectangular ideal luminance waveform to real luminance data acquired through VISTO.

Concerning sensory response functions, we assumed that a luminance or contrast change issuing from a display device triggers a sensory response in the visual system which, informally speaking, is a neurally based, temporally blurred version of the stimulus's luminance profile. The sensory-response function—sensory-response magnitude as a function of time since stimulus onset—is often modeled as a linear system by convolving the display device's stimulus luminance profile with the visual system's impulse-response function, and scaling the result by stimulus contrast (e.g., [4,30]) which may vary in its temporal properties, and specifically in the low-pass temporal decay parameter which usually range from 3 to 9 ms [4,16]. In turn, sensory response functions form the bases for more complex cognitive operations, which result in performance measures.

To determine the effect of different luminance profiles on a typical performance measure, we assumed a theory that has been confirmed to a large degree of precision in accounting for effects of various low-level stimulus attributes, such as stimulus contrast and duration, on memory performance for digit strings (e.g., [4]), line drawings [14], black-and-white pictures of objects [16], and random forms [9]. Very briefly, this theory makes the following assumptions. First a visual stimulus triggers a sensory-response function as described above. Second, there is a sensory threshold, characterized in units of stimulus contrast, such that stimulus information acquisition takes place only while sensory-response magnitude is above threshold. Third, stimulus information is acquired at an instantaneous rate that is proportional to the product of (a) the degree to which the sensory response exceeds threshold and (b) the proportion of yet-to-be-extracted stimulus information. Fourth, performance is equal to proportion of acquired information. A consequence of these assumptions is that performance is predicted to be related to the above-threshold area under the sensory-response function by an exponential equation of the form,

$$p=1-e^{\text{Ax}/c}$$

where p is the predicted performance, Ax is the above-threshold area and c is a scaling constant.

**Fig. 4:**
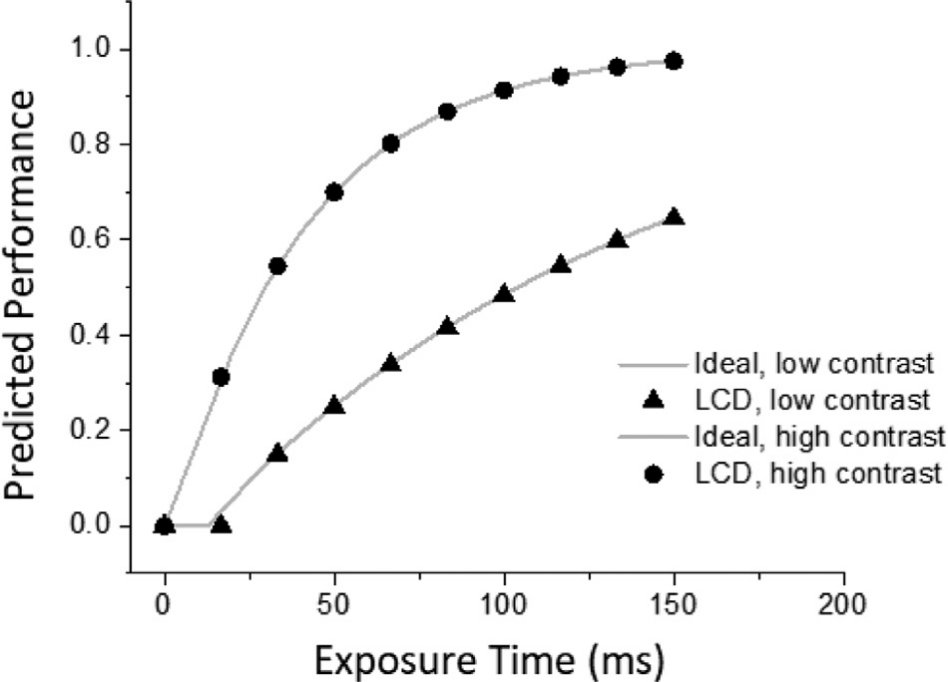
Predicted performance for LCD and ideal display devices, for high (0.10) and low (0.03) contrast levels.

We generated theoretical predictions, assuming a typical threshold value of 0.02, for durations ranging from 16 ms to 150 ms for two contrast levels: 0.10 (“high”; that is well above threshold), and 0.03 (“low”; that is, close to threshold), and for a low-pass temporal decay parameter of the sensory-response function of 3 ms. The results are shown in Fig. 4. Even for the shortest-duration stimuli, no performance differences are evident between ideal and real luminance waveforms. Thus, it seems safe to conclude that theoretical predictions involving performance (e.g., proportion correct or d’) are met by an LCD monitor with a luminance waveform similar to that reported in Fig. 3.

### Determination of exposure time

In everyday laboratory work, researchers are interested in determining exact exposure times for their stimuli. Here we describe how to use VISTO for measuring the exposure time of a stimulus while it is presented, without requiring additional instrumentation.

### Data Acquisition and Procedure

**Fig. 5:**
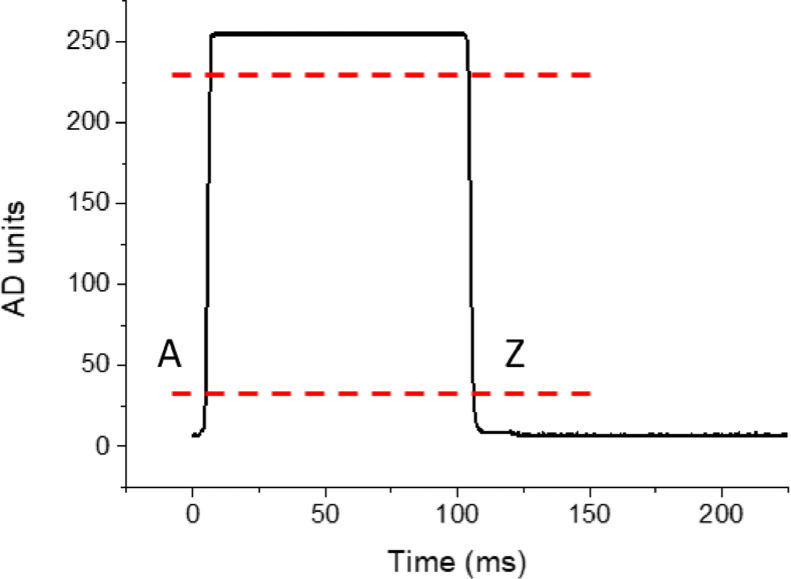
Schematic representation of event coding during a trial, for a 100 ms stimulus. Red dashed lines represent calibration thresholds. Region above the upper red line is the “white” region, while region below the lower red line is the “black” region, and points A and Z define the exposure time from black to white, and vice-versa.(For interpretation of the references to color in this figure legend, the reader is referred to the web version of this article.)

**Fig. 6:**
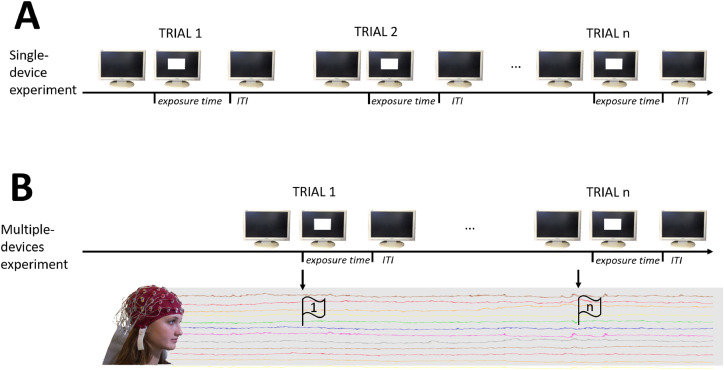
Schematic representation of experimental settings in which a single device is involved, and in which exposure time of bright patches and blank ITI alternate; and of multiple-devices settings in which more than one device (in this example, EEG) must be synchronized. Note that, while in EEG trial 1 the visual onset and the EEG trigger are in sync with each other, in trial n the EEG trigger anticipates picture onset.

To establish a criterion to determine exposure time, we coded each trial as a sequence of transitions from black to white, and then to black again (Fig. 5). To do this, we first calibrated the device to determine the luminance values (in arbitrary AD units) for white and black displays, and then set thresholds to code each luminance state as black, white, or in between (red lines in Fig. 5). Below, there are specific instructions on how to calibrate VISTO; here, we specify the additional detail that, compared with the calibration value, a threshold is calculated which is 10% of the calibration range (from the maximum black value to the minimum white value). For instance, if the calibration value for black is 9-10 AD units and the calibration value for white is 250-254 AD units, then the range is 250 – 10 = 240, and the threshold will be set at 240 ⋅ .10 = 24 AD units. This threshold value will be used to determine the point of transition from black to white (A point in Fig. 5), corresponding to the maximum calibration value for black + the threshold; 10 + 24 = 34. In an experiment in which bright and dark patches alternate (Fig. 6A), exposure time was defined as the time between the change from black to white (point A, see Fig. 5), and the return from white to black (point Z). The remaining time (between the black onset Z, and the black offset A of the next picture) was defined as the inter-trial interval (ITI). The visual signal is acquired at 2000 Hz and 8 bits resolution, and computations are done and displayed on the built-in VISTO display.

**Fig. 7:**
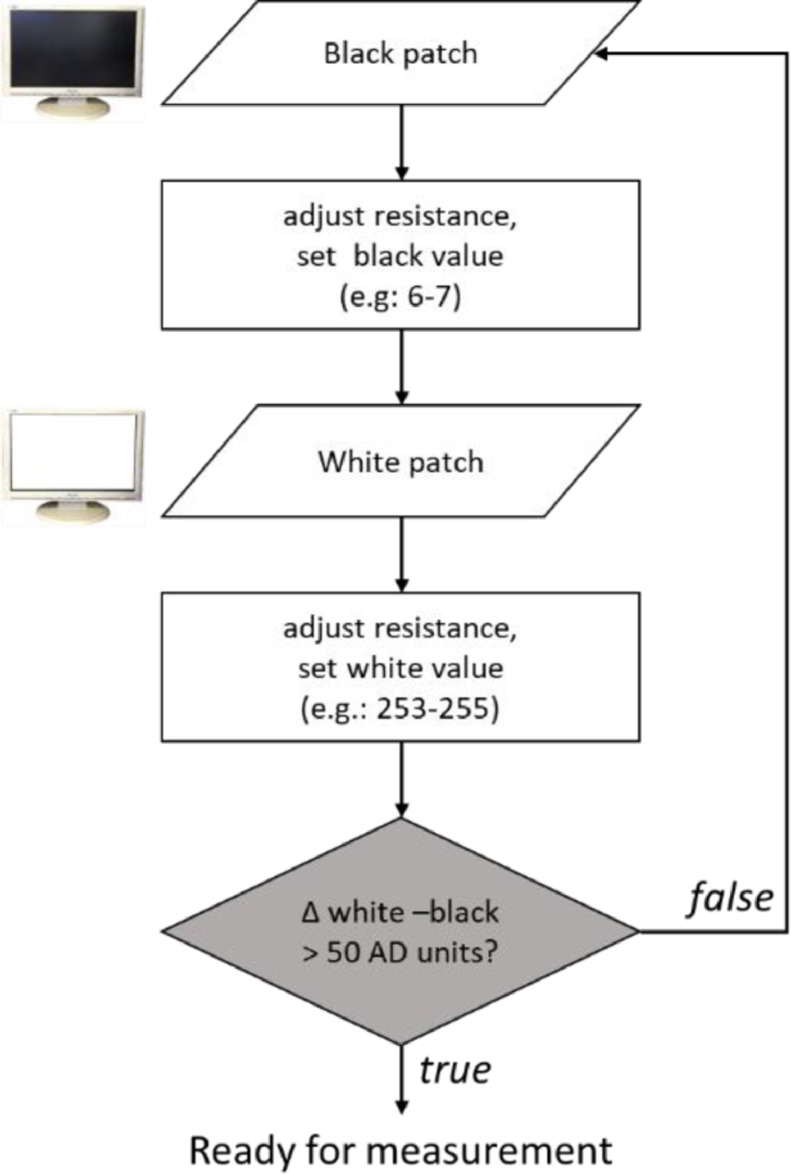
Flowchart of the calibration procedure.

*Calibration*. The aim of the calibration phase is to adjust the data acquisition range to the white-black range of the specific display device used. For this reason, a black and a white patch must be presented, and VISTO uses these patches to define the “white” and “black” thresholds that will be used to calculate exposure time (Fig. 5). In the calibration phase, black and white patches must be presented onscreen, and the device is calibrated to get a “black” and “white” value as different as possible from each other. To this end, variable resistances are adjusted to set optimal levels for “black” and “white” stimuli (flowchart in Fig. 7). In this phase, the photodiode is attached to the monitor, and a screen (first dark and then light) is presented. When the calibration button for black/white is pressed, the minimum and maximum luminance value observed in a 100 ms time interval are displayed; in this phase, low variability and no floor or ceiling values are desirable (e.g. 6–7 AD units for black screen, and 253-255 AD units for white screen). After both black and white values have been calibrated, if the range of the values is acceptable (i.e., separated by at least 50 AD units, corresponding to a minimum threshold of 5 AD units), the experimenter can proceed to the testing phase.

Exposure Time Measurement Procedure

**Fig. 8:**
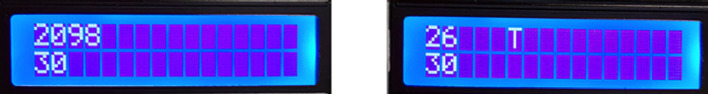
VISTO display for an experiment where black and white displays alternate (left; experimental procedure in Fig. 6A), and when white displays are preceded by a trigger (right; experimental procedure in Fig. 6B). In both cases, the white display is presented for 30 ms. In the experimental paradigm shown on the left, the white display is preceded by a 2098 ms black display. In the experimental paradigm shown on the right, a trigger is presented 26 ms prior to the white display.

1) Installation steps are identical to the “luminance waveform acquisition” procedure, except that the “ExpTime.ino” (Supplementary Code B) file should be loaded to VISTO, and that no additional software other than Arduino Ide is required. If the file is loaded correctly, then the device will display the message “EXPTIME Ready!”. Once this file has been loaded, VISTO does not need to be connected to a PC anymore except if the experimenter wants to save the timing results on a text file.
2) Run the calibration, as described above.
3) Run the experiment, consisting of alternating black and white patches, which can optionally include triggers during the black (no stimulus) screen. After each transition from black to white and vice-versa, exposure time will be presented on the VISTO built-in screen, along with the intertrial interval (ITI) or with the difference between the onset of the white patch and the trigger on the parallel port (see Fig. 8). Moreover, the same values are sent in text format to the USB port, where they can be captured as described for luminance profiles. Along with intertrial/trigger time and exposure time, absolute times are acquired for beginning of black, ending of black, beginning of full white, ending of full white, and trigger onset.

### Signal delay and synchronization between devices

An additional timing problem occurs when measures are to be time-locked to the onset of a visual stimulus (e.g., ERPs or eye tracking). The problem occurs because there are device-dependent delays between video signal and display-device response; as shown by Brainard et al. [3], with an LCD projector this delay may be as much as 12 ms. Because of several issues, including those mentioned above (non-real-time systems, time needed to load pictures from the hard drive and to prepare the visual stimulus for display, latency in video card and LCD response, etc.), it is not straightforward to assume that the intended onset and offset times are respected. Fig. 6B, for instance, displays a schematic representation of an EEG recording session, in which event triggers (downward arrows) are sent for each trial, and these triggers are used to tag the EEG signal and mark the onset of each specific visual stimulus. While for trial 1 the event onset (vertical tick) and the EEG trigger (downward arrow) are well synchronized, for trial n there is a visible delay, and the EEG trigger anticipates the onset of the visual stimulus. An unpredictable delay between triggers and actual onsets can be detrimental for time-locked responses, including EEG/ERPs, eye movement tracking, and even for response-time measurement. If this condition does not hold, a random jitter is introduced into the data. Concerning ERPs, this results in a smearing of ERP waveforms, as discussed by Kappenman & Luck [10, p. 12]. More complex settings such as using virtual reality devices, wearable activity trackers, or EEG hyper-scanning pose similar problems and require an additional setting and testing phase prior to data collection.

Concerning synchronization, VISTO allows for testing the delay between triggers sent through the parallel port, and the onset of a visual pattern. When a trigger is detected, the delay between this signal and the picture onset will be automatically calculated and displayed, followed by a “T” (Fig. 8), to indicate that the value refers to the distance between a “trigger” and the visual stimulus.

### Tests of synchronization

When the device is used in combination with a triggering signal, it is critical to test the reliability of the relative times between the trigger signal and the veridical onscreen stimulus. Professional presentation software provides extensive logging capabilities, however the variability in LCD monitor responses is not reflected in software logs. For this reason, we created an experiment in which we compared VISTO timings to software timings. In this simple experiment, a total of 100 stimuli (white patches, 100 ms exposure time) were preceded by a trigger sent from the presentation PC. Additionally, a second photodiode was attached to a BIOPAC amplifier, which recorded continuously luminance changes, to perform a simultaneous acquisition of veridical signals measured through the BIOPAC-connected photodiode, software logs, and VISTO relative timings. We programmed these experiments on E-Prime 2.0 ([24]; Intel Q963/Q965 Graphics Controller) and Presentation (Neurobehavioral Systems, Inc., Berkeley, CA; Integrated Video Card Intel Q45/Q43 Chipset), and on both platforms the experiment structure was the same, consisting of,

• A black intertrial interval.
• A trigger code, temporally aligned with the monitor vertical sync, sent from the presentation PC through the parallel port to VISTO and the digital BIOPAC input.
• A white stimulus patch, which is synchronized to the vertical refresh of the monitor and is programmed to be presented for 100 ms (6 frames on a 60 Hz monitor).

**Fig. 9:**
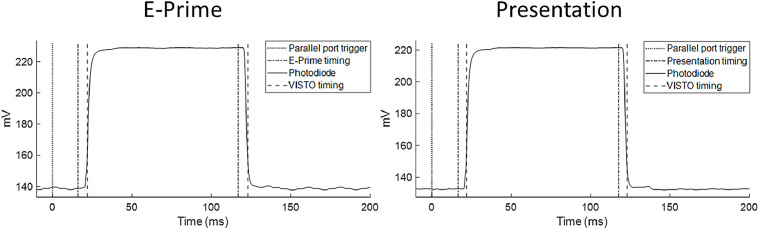
Comparison of event timings by VISTO and E-Prime (left) and Presentation (right). The parallel port input coming from the presentation PC is shown as a dotted vertical line. The veridical stimulus signal (solid line: screen luminance recorded through a second photodiode acquired with a BIOPAC system) is plotted together with the event markers indicated by presentation software (dashed-dotted lines) and by VISTO (dashed lines).

We tested both programs on a monitor with 100% brightness and contrast, and on the same monitor with 50% brightness and contrast. For each level of brightness/contrast we calibrated VISTO as described above. Both the VISTO and the BIOPAC photodiodes were attached to the upper end of the monitor, to catch the beginning of screen redraw. Data from a representative trial, including photodiode signal, software logs and VISTO timings, are reported in Fig. 9; in Table 1, the overall results are reported. We note three main results. First, the delay between the trigger and the stimulus was not zero because, in the experimental scripts, both the trigger and the white patch required the system to wait for the next vertical refresh, creating a minimum trigger-stimulus delay that was equal or larger than the duration of a monitor refresh (16.67 ms on a 60 Hz monitor). Second, both software logs and VISTO reported a standard deviation lower than 0.5 ms for all experimental objects, testifying to the high temporal accuracy and, in this particular experiment, to the absence of temporal jitter which could be worrisome e.g., for ERP analysis. Third and most important to the present device, there were up to about 7 ms differences in the absolute timing, e.g. in the trigger-stimulus delay, between software logs and VISTO. More specifically, while software logs reported a smaller delay (about 16 ms, approximately corresponding to the monitor refresh rate), VISTO reported larger values (about 23 ms), possibly because of the time needed by the monitor to begin stimulus display and raise the brightness of the white patch. Veridical stimuli align well with the VISTO timing.

**Table 1:** Mean and SD (in ms) of trigger-stimulus delay, and of exposure time, as reported by software logs and by VISTO. In the last two columns, the difference (mean and SD, in ms) between VISTO measures and the software logs are shown.

		SOFTWARE LOG		VISTO		VISTO - SOFTWARE DIFFERENCE	
BRIGHTNESS/ CONTRAST	OBJECT	MEAN	SD	MEAN	SD	MEAN	SD
50%	Trigger-Stimulus Delay	16.00	0.14	22.81	0.42	6.81	0.42
100%	Trigger-Stimulus Delay	16.00	0.14	22.54	0.50	6.54	0.50
50%	Exposure Time	100.43	0.50	100.00	0.00	-0.43	0.50
100%	Exposure Time	100.42	0.50	100.37	0.49	-0.05	0.66
50%	Trigger-Stimulus Delay	16.70	0.01	22.33	0.47	5.63	0.47
100%	Trigger-Stimulus Delay	16.70	0.00	22.00	0.00	5.30	0.00
50%	Exposure Time	100.00	0.00	100.19	0.39	0.19	0.39
100%	Exposure Time	100.00	0.00	100.37	0.49	0.37	0.49

While both software logs and VISTO are similarly stable in their measurements, VISTO can report the timing of the actual rise in stimulus luminance, therefore providing a better temporal anchoring for paradigms in which it is critical to assess the exact moment in which a stimulus becomes visible. Moreover, when stimuli cover only a portion of the screen (e.g., small stimuli positioned in the center of the monitor; e.g., [15]), VISTO can accurately report the latency of stimulus presentation at a specific location.

## Conclusion

Motivated by several impediments to trustable timing, we have presented a low-cost, open-source device that can be used to measure luminance profiles and to calculate precise exposure times. VISTO can be used at the time of setting up an experiment, so that an experimenter can ensure that the experimental devices behave as desired; at the same time, it can detect and log event markers during an experiment, which can aid data analysis. Moreover, VISTO can be calibrated to fit monitors of different luminance range, and software changes can be achieved by modifying the Arduino code.

Being able to manipulate the timing of a visual stimulus is crucial in several psychological domains. We form impressions quickly [2], respond to briefly presented emotional stimuli [5], and are extremely rapid in categorizing visual object and scenes [29] and in judging the probability of risk [23]. Moreover, as technology evolves, more experiments can be run outside of the standard laboratory. For instance, it is possible to run experiments on portable devices such as smartphone or tablets, possibly in more real-world settings. However, these devices may also present problems similar to standard monitors. In other words, even if it is assumed that the presentation software will take care of the intervening variables (e.g., running the experiment with a higher priority compared to other software), difficulties may arise due to delay in the video chip, in the internal clock of the display, or on the technology used to create images. There is no general solution to this issue, other than measuring the exact timing of the to-be-presented visual stimuli.

The data collected here indicate that once reliable timing has been achieved, sensory response functions as well as predicted performance were essentially identical assuming actual luminance waveforms and ideal rectangular waveforms. We did this comparison based on functions which have been shown to fit diverse kind of situations such as memory for digits, drawings, real world pictures, and abstract shapes [4,9,14,16]. What is crucial here is that exposure time is critical to determine sensory response function and in turn performance, while slight differences in rising or falling luminance slope do not play major roles. If expected timing is achieved, then information accumulation will be similar for ideal as well as for actual display devices. If they are not however, e.g., if an expected 50 ms stimulus is actually shown for 33 or 66 ms, then there will be large departures from the ideal curve and, which will lead to large and unpredictable differences in visual processing.

## Limitations and future directions

We described here a simple device for checking the timing of visual stimuli. The wide availability of the materials makes VISTO easy to assemble and to distribute, with benefits in terms of experimental control. There are, of course, limitations in terms of what VISTO can do, for instance, extending VISTO to a more complex setting in which auditory and visual stimuli must be synchronized, or monitoring the onset of visual stimuli and sending timing codes to an acquisition machine, for which more specific tools may be available (e.g., [7], [27]; Trigger Station by BrainTrends, Ltd.). Here we used VISTO to describe the properties of the rising and falling luminance waveform for an alternation of white/black patches, however the same approach can be used to measure the visualization of stimuli (e.g., pictures of natural scenes, fonts, etc.), which have a high between-stimulus variability in brightness that can influence luminance waveforms. As the parallel port is an outdated technology, in the future the input of VISTO can be used with other trigger types (e.g., digital outs I/O cards) that can send a 5 V signal. Moreover, the general scheme which is presented here can be easily modified to change the transducer characteristics (e.g., a different photodiode which is more sensitive to specific light wavelengths, e.g. for animal research) or type (e.g., a microphone), thus serving the needs of different types of laboratories.

## Declaration of Competing Interest

The authors declare that they have no known competing financial interests or personal relationships that could have appeared to influence the work reported in this paper.

## Appendix Supplementary materials

Image, application 1

Image, application 2
