# Corrigendum to “VISTO: An open-source device to measure exposure time in psychological experiments”

**DOI:** 10.1016/j.mex.2021.101467

**Published:** 2021-07-22

**Authors:** Andrea De Cesarei, Michele Marzocchi, Geoffrey R. Loftus

**Affiliations:** a Department of Psychology, University of Bologna, Italy; b Department of Psychology, University of Washington, United States

In paper “VISTO: An Open-Source Device to Measure Exposure Time in Psychological Experiments”, a device that measures the onset of experimental visual stimuli is described. This device acquires luminance information through a light sensor BPW42, and then records luminance waveforms or onset times. Throughout the paper however, sensor BPW42 was erroneously described as a photodiode, while it is a phototransistor. Moreover, the wiring scheme in Fig. 1 was incomplete.

**Fig. 1:**
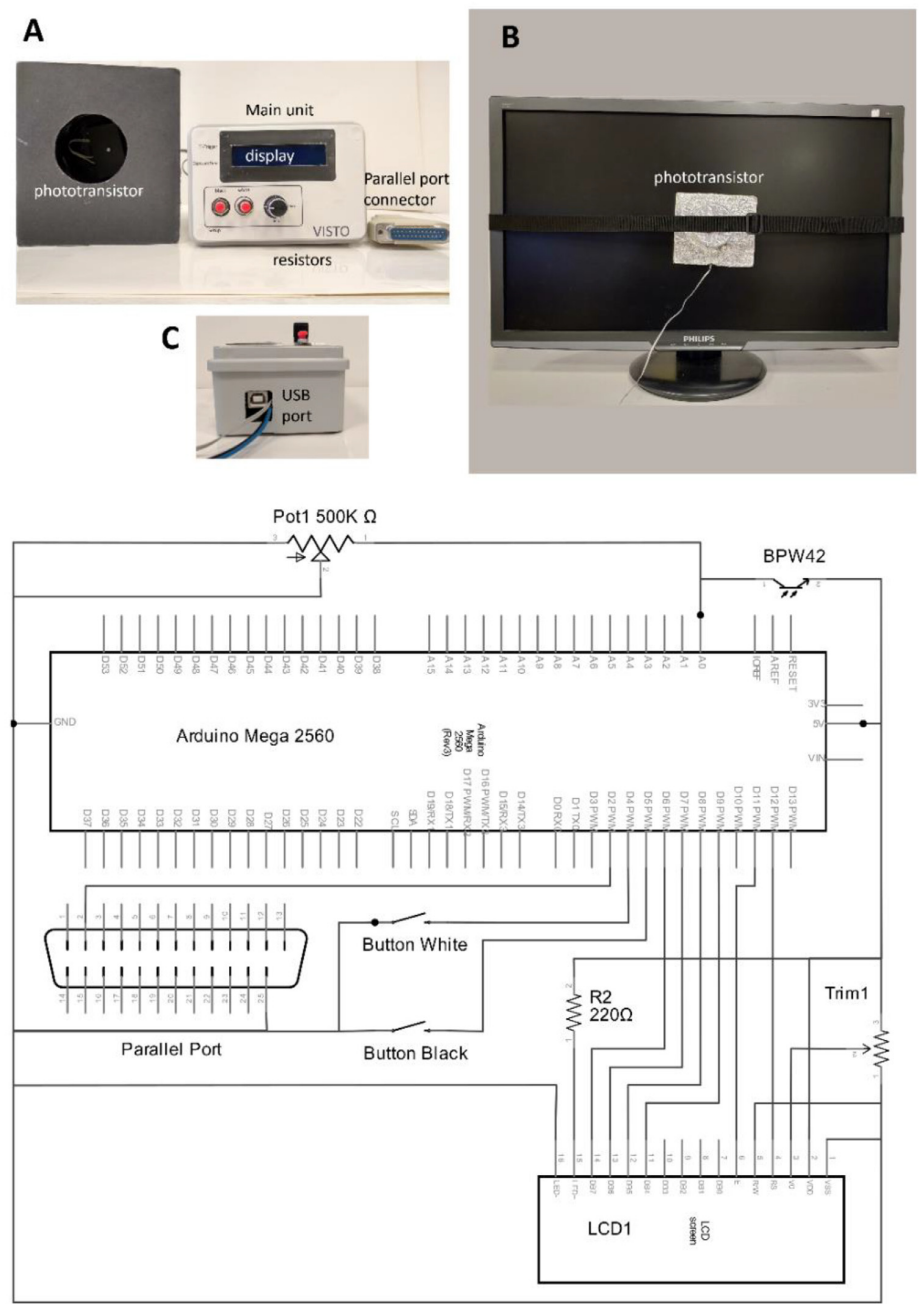
Upper panel, A-C: VISTO main components. Lower panel: diagram of the electronic circuit.

In the revised Fig. 1, sensor BPW42 is correctly labeled as a phototransistor, and the correct wiring of all components is reported.

The authors would like to apologize for any inconvenience caused.

